# Development of Fault Similar Material for Model Test of Fault Water Inrush Disaster

**DOI:** 10.3390/ma18163745

**Published:** 2025-08-11

**Authors:** Zhipeng Li, Deming Wang, Kai Wang, Qingsong Zhang, Lianzhen Zhang, Yang Gao, Yongqi Dai

**Affiliations:** 1 School of Transportation and Civil Engineering, Shandong Jiaotong University, Jinan 250357, China; lizhipeng@sdjtu.edu.cn (Z.L.); daiyongqisdjtxy@163.com (Y.D.); 2 Shandong High-Speed High-Tech Investment Co., Ltd., Jinan 250102, China; wakai9958@163.com; 3 Research Center of Geotechnical and Structural Engineering, Shandong University, Jinan 250100, China; zhangqingsong@sdu.edu.cn; 4 College of Pipeline and Civil Engineering, China University of Petroleum, Qingdao 266580, China; zhanglianzhen@upc.edu.cn; 5 Key Laboratory of Structural Health Monitoring and Control, Shijiazhuang Tiedao University, Shijiazhuang 050043, China; gaoyang_sjz@163.com

**Keywords:** model test, fault water inrush, similar material, mix ratio, physical–mechanical parameters

## Abstract

The applicability of similar materials is a key factor affecting the results of geomechanical model tests. In order to investigate the multi-physical field evolution mechanism of surrounding rocks during water inrush disasters in tunnels crossing fault zones, based on the similarity theory of geomechanical model tests, the physical–mechanical parameters of a prototype rock’s mass were first analyzed for similarity, and the target values of similar materials were determined. Secondly, using sand as coarse aggregate, talcum powder as fine aggregate, gypsum and clay as binders, and Vaseline as a regulator, a fault-simulating material suitable for model tests was developed through extensive laboratory experiments. Finally, with material deformation characteristics and strength failure characteristics as key control indicators, parameters such as uniaxial compressive strength, permeability coefficient, unit weight, and elastic modulus are synergistically regulated to determine the influence of different component ratios on material properties. The experimental results show that the uniaxial compressive strength and permeability coefficient of similar materials are mainly controlled by gypsum and Vaseline. Cohesion is mainly controlled by clay and Vaseline. The application of this similar material in the model test of the tunnel fault water inrush disaster successfully reproduced the disaster evolution process of fault water inrush, meeting the requirements of the model test for similar materials of faults. Furthermore, it provides valuable guidance for the selection of similar materials and the optimization of mix proportions for fault disaster model tests involving similar characteristics.

## 1. Introduction

Fault-zone rock masses are characterized by developed fractures, loose and fragmented structures, and high-pressure water-rich conditions. Tunnels crossing faults are extremely prone to inducing water inrush disasters [1,2,3], which endanger the safety of construction personnel, cause construction delays, and threaten the ecological environment of the tunnel site and the long-term operation safety of the tunnel. Studying the variation laws of each physical field in the surrounding rock during the occurrence of water inrush disasters is of great significance for guiding tunnel design, construction, and risk avoidance.

Currently, the research methods for tunnel water inrush mainly include theoretical calculations [4,5,6], numerical simulations [7,8,9], and model tests [1,10,11,12,13]. However, the complex formation mechanism and occurrence conditions of faults make the evolution process of water inrush disasters extremely complex, with significant coupling effects of physical field parameters. It is difficult to conduct a comprehensive analysis and research relying solely on theoretical and numerical calculations. As a critical research method, geomechanical model testing accurately reproduces the entire evolution process of tunnel water inrush disasters, with easily controllable test conditions and variables, which play a critical role in addressing underground engineering disaster mechanisms. Reasonable and reliable similar materials have an important impact on the results of model tests. Fault rock masses have characteristics such as high porosity, high softening coefficients, and easy disintegration when exposed to water. The selection and proportioning of material components are key factors in fault similarity simulation.

Dai et al. [14] used talcum powder, gypsum, and liquid paraffin as raw materials, optimizing the ratio through orthogonal tests to successfully simulate the permeability characteristics of tunnel rock masses. Ko et al. [15] used Portland cement and gypsum as binders, combined with mica powder filling, to construct similar materials that can characterize the crack propagation behavior of rocks. Zan et al. [16] used a granular mixture of similar materials composed of quartz sand, barite powder, and gypsum, adjusting the hydraulic properties of the similar materials through silicone oil, liquid paraffin, industrial glycerin, and petrolatum gel, which were successfully applied to a seepage model test of tunnel excavations in Xi’an Metro Line 9. Shemenda et al. [17] used paraffin, mud, and other materials as cementing materials, and river sand and gypsum were used as aggregates to make similar rock materials. Zhang et al. [18] derived the similarity criteria for permeability coefficient, seepage velocity, flow velocity, and seepage pressure under high ground stress and high groundwater conditions based on the similarity theory of hydraulic coupling, and they developed similar materials using iron ore concentrate powder, barite powder, and quartz sand as aggregates, white cement as a cementing agent, and silicone oil to regulate the hydraulic properties of the materials. The above scholars’ inorganic cementing materials, with Portland cement and gypsum as the core, have the characteristics of low cost and high temperature resistance, but they are relatively brittle and have insufficient long-term stability.

Xu et al. [19] developed similar materials composed of quartz sand, barite powder, silicone oil, Vaseline, and paraffin, adjusting the ratio through orthogonal tests to configure the surrounding similar rock materials that can meet the requirements of uniaxial compressive strength, elastic modulus, gravity, and bondability. These scholars introduced organic components such as nano-clay and silicone oil, significantly improving the toughness of materials. Liu et al. [20] developed a new type of fluid–solid-coupling similar material, which was mixed with river sand, calcium carbonate, talcum powder, white cement, Vaseline, and anti-wear hydraulic oil, verifying its feasibility through physical simulation tests in coal mine surface water inrush events. Guo et al. [21] developed similar fault materials using mountain sand, gravel, and red clay as raw materials based on the fluid–solid coupling similarity theory and carried out a large number of matching tests, revealing the evolution process of water inrush in deeply buried tunnels. Wu et al. [22] developed fluid–solid coupling similar materials (SCVO) using sand, barite powder, and talcum powder as aggregates; cement and Vaseline as cementing agents; and silicone oil as a regulator, and they successfully applied them to a geomechanical model test of the Qingdao Jiaozhou Bay Submarine Tunnel.

It can be seen from the above that although certain progress has been made in the development of fault similar materials, there is still the problem of poor similar material simulation. At present, very few similar materials can simultaneously meet the physical, mechanical, and hydrogeological properties of faults, and targeted regulation cannot be performed on the characteristic parameters of different simulation objects. Based on the above research results and similarity theory, this paper uses sand, talcum powder, gypsum, Vaseline, and clay as the main components to develop a fault similar material that meets physical, mechanical, and hydrogeological properties through mix ratio tests, and we successfully applied it in a model test of a tunnel fault water inrush disaster.

## 2. Methods and Specimen Preparation

### 2.1. Similarity Principle

When designing similar model tests, the similar conditions should be determined according to the similarity of the research object: that is, the geometric dimensions, loads, boundary conditions, material density, strength, deformation characteristics, and hydrogeological characteristics of the prototype and the model follow the similarity principle [23]. The similarity scale (C) refers to the ratio of physical quantities with the same dimension between the prototype (P) and the model (M). Based on dimensional analysis and the basic equations of elastic mechanics, combined with the fluid–solid coupling theory, the following similarity relationships are derived.

(1) According to dimensional analysis, the similarity scales of physical quantities with the same dimension are equal, and the similarity scale with a dimension of one is equal to 1; we then have the following:

(1) $$C_{\mu }=C_{\varepsilon }=C_{\varphi }=1,$$

(2) $$C_{\sigma }=C_{\sigma_{c}}=C_{\sigma_{t}}=C_{E}=C_{c},$$

(2) The similarity relationship derived from the equilibrium equation is as follows: Prototype equilibrium equation:

(3) $${\left(\sigma_{ji,j}\right)}_{p}+{\left(f_{i}\right)}_{p}=0,$$

Model equilibrium equation:

(4) $${\left(\sigma_{ji,j}\right)}_{m}+{\left(f_{i}\right)}_{m}=0,$$

According to the definition of similarity scales, substituting the similarity scales of stress, geometry, and volume force $C_{\sigma },C_{L},C_{f}=C_{\gamma }$ into Equation (3) gives

(5) $$\frac{C_{\sigma }}{C_{L}}{\left(\sigma_{ji,j}\right)}_{m}+C_{\gamma }{\left(f_{i}\right)}_{m}=0,$$

Comparing Equations (4) and (5) gives

(6) $$\frac{C_{L}C_{\gamma }}{C_{\sigma }}=1,$$

(3) In addition, according to the geometric equation, physical equation, stress boundary condition, and displacement boundary condition, the following similarity relationships can be derived:

(7) $$\frac{C_{\delta }}{C_{\varepsilon }C_{E}}=1,$$

(4) Based on the mathematical model of fluid–solid coupling in homogeneous and continuous media, the similarity theory of fluid–solid couplings is derived, and the corresponding similarity relationships are obtained as follows:

(8) $$C_{K}=\frac{\sqrt{C_{L}}}{C_{\gamma }},$$

In Equations (1)–(8), we have the following: γ is the unit weight; L is the geometric dimension; δ is the displacement; E is the elastic modulus; σ is the stress; $\sigma_{t}$ is the tensile strength; $\sigma_{c}$ is the compressive strength; ε is the strain; c is cohesion; φ is the internal friction angle; μ is Poisson’s ratio; K is the permeability coefficient. For similarity scales, $C_{\mu }$ is the Poisson ratio similarity scale; $C_{\varepsilon }$ is the strain similarity scale; $C_{\varphi }$ is the friction angle similarity scale; $C_{\sigma }$ is the stress similarity scale; $C_{\sigma_{c}}$ is the compressive strength similarity scale; $C_{\sigma_{t}}$ is the tensile strength similarity scale; $C_{E}$ is the elastic modulus similarity scale; $C_{c}$ is the cohesion similarity scale; $C_{L}$ is the geometric similarity scale; $C_{\gamma }$ is the unit weight similarity scale; $C_{K}$ is the permeability coefficient similarity scale.

### 2.2. Determination of Similar Material Parameters

In order to accurately simulate the mechanical behavior and engineering response of a faulted rock body in an actual project, materials similar to the actual faulted rock body in terms of mechanical properties are developed. In this study, the Zhongjiashan Tunnel Project of the Quannan Expressway in China is used as the background. Based on the geological investigation report of the project and the core samples taken from the site, it is clarified that the lithology of the fault-filling medium is mainly muddy, fully weathered shale, which contains fault clay and fault breccia, and it is characterized by a loose structure with alternating soft and hard layers; plasticity is exhibited when the water content is high, and cracking is observed when water loss occurs. The parameters of this prototype faulted rock mass are shown in Table 1.

To simulate the characteristics of the fault rock body and related engineering problems, the similarity ratio is determined based on the similarity principle, which is based on the requirement of realism of the model test. Here, $C_{L}=60$, $C_{\gamma }=1$, $C_{\sigma }=60$, $C_{E}=60$, $C_{K}=\sqrt{60}$, $C_{c}=60$, and $C_{\varphi }=1$, and the calculations are obtained to simulate the similar material of each target parameter, as shown in Table 1.

### 2.3. Material Preparation

**Figure 1:**
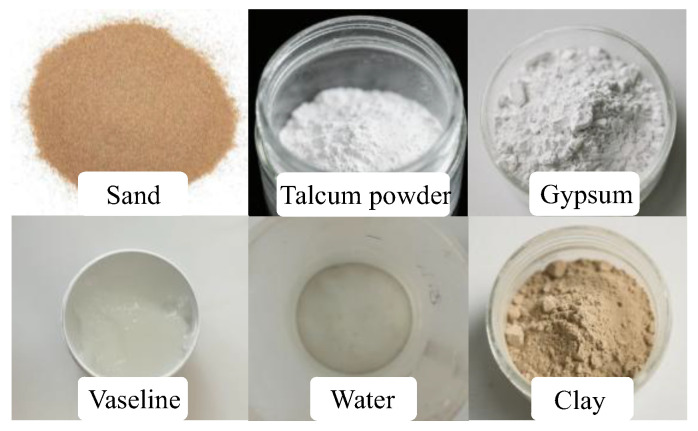
Fault similar material composition.

The prototype fault rock mass in this test contains fault clay and fault breccia, which are characterized by a loose structure with alternating soft and hard layers, and plastic behavior is observed when the water content is high, with cracking occurring upon water loss. In this study, gypsum and clay were used as binders; Vaseline as a regulator; and sand and talcum powder as aggregates, and an appropriate amount of mixing water was used to develop a new type of fault similar material. The material components are shown in Figure 1.

**Figure 2:**
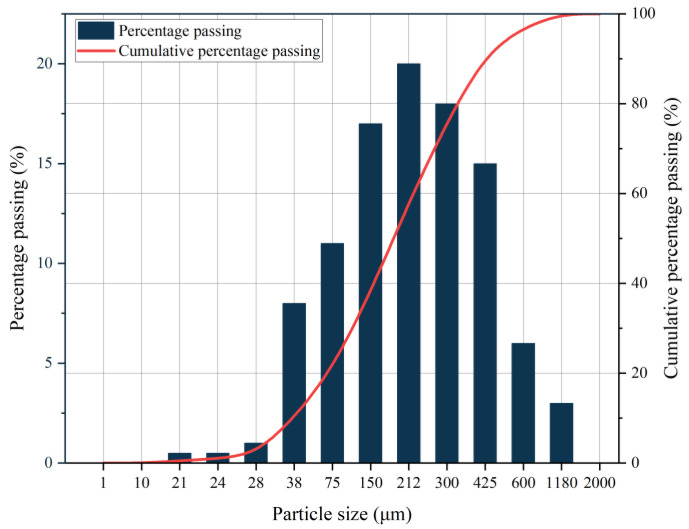
Sieving curves for sand.

To meet the requirements of the physical, mechanical, and hydrogeological properties of similar materials, the particle size of sand is <2 mm, and the particle size distribution diagram of the sand is shown in Figure 2, the gypsum used is building gypsum with a fineness of 600 meshes, the clay selected is silica–aluminate clay, and the fineness of talcum powder is 1250 meshes, with the main component being Mg_3_Si_4_O_10_(OH)_2_, which is a kind of water-containing magnesium silicate mineral that accounts for more than 90% of the talc.

**Figure 3:**
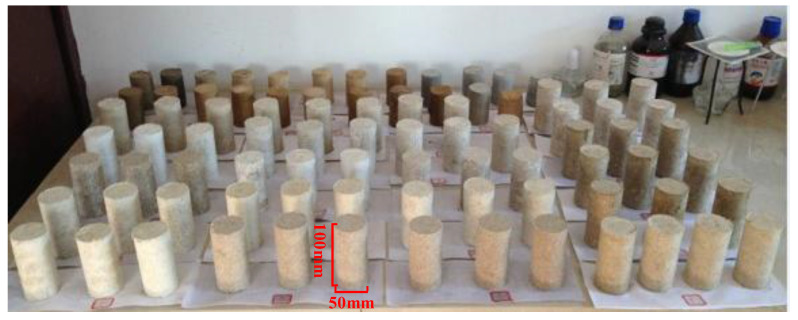
Similar material specimen.

Specimens of the similar material were prepared using molds with dimensions of ϕ50 mm × 100 mm. The coarse and fine aggregates, binders, and regulators were accurately weighed according to mass. The coarse and fine materials and clay were mixed and stirred evenly, water was added, followed by Vaseline, and the mixture was thoroughly stirred. The mixture was then placed into the mold and compacted in multiple layers to ensure uniformity in both the upper and lower parts of the specimen. After demolding, the specimens were cured at room temperature. The specimens of the similar material are shown in Figure 3.

### 2.4. Test Methods

**Figure 4:**
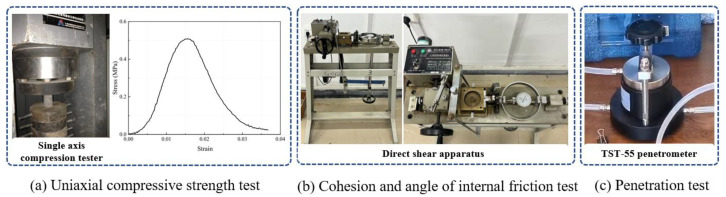
Schematic diagram of the test method.

The performance test of similar materials includes a test [24] of the basic mechanical parameters and hydrogeological parameters of the materials. The test of basic mechanical parameters includes the test of uniaxial compressive strength $\sigma_{c}$, elastic modulus E, cohesion c, and internal friction angle φ. The test of hydrogeological parameters includes the disintegration and permeability coefficient K of the materials. These parameters are tested as shown in Figure 4. To test the performance of different ratios of similar materials and obtain the influence of each factor on the material parameters, three specimens were made for each parameter of each ratio.

(1) Determination of Uniaxial Compressive Strength and Elastic Modulus of the Materials

For standard specimens with dimensions of ϕ50 mm × 100 mm, a microcomputer-controlled electro-hydraulic servo testing machine was used to perform uniaxial compression tests, as shown in Figure 4, to determine the stress–strain curve of the material specimens during the compression process, as shown in Figure 4. The slope of the elastic stage of the curve is the elastic modulus.

(2) Determination of Material Cohesion and Internal Friction Angle

The internal friction angle and cohesion of the fault similar material were determined using a strain-controlled direct shear instrument (Naijiuweiyen Technology Co., Beijing, China). First, the similar material was pressed into direct shear specimens with dimensions of ϕ61.8 mm × 20 mm using a special mold. After demolding, the specimens were placed in a constant-temperature and constant-humidity box for standard curing (temperature = 20 ± 2 °C; relative humidity ≥ 95%) [25] for 7 days, and natural air drying took place for 3 days. Subsequently, the cured specimens were placed in the shear box of the direct shear instrument, with permeable stones above and below the specimens. After the specimens were properly installed, loading was performed. Four specimens were used for each experiment, and direct shear tests were conducted under vertical pressures of 100, 200, 300, and 400 kPa at a shear rate of 0.8 mm/min. The specimens were sheared to failure within 4–5 min, and the maximum readings on the dial gauge were recorded under each level of vertical load. The shear strength of the material was calculated using the following formula:

(9) τ=σtanφ+c,

In the formula, τ is shear stress, and σ is normal stress.

The c and φ values of the fault similar material specimens with typical mix ratios are shown in Table 2.

(3) Material Hydro-Physical Property Testing

① Water Disintegration Test

The fault-filling medium, when exposed to water, experiences partial dilution, softening, or dissolution of its cementing agents, leading to a reduction in cohesion and an increase in looseness. As the water–rock interaction continues, this process ultimately causes the weakening and disintegration of the fault-filling material, resulting in water surges. To simulate this characteristic of fault similar materials, specimens of fault similar materials are cured at room temperature for 7 days before undergoing a water disintegration test. The disintegration times of specimens with typical mix ratios are shown in Table 3.

② Permeability Coefficient K Test

Permeability, as another important indicator of the hydro-physical properties of similar materials, can be characterized by the permeability coefficient. The higher the permeability coefficient, the stronger the permeability. Considering that the permeability coefficient of geotechnical materials is generally small, typically in the range of 10^−7^–10^−3^, and that various interference factors make it difficult to ensure high measurement accuracy, a TST-55 permeability meter (NAIJIUWEIYEN TECHNOLOGY CO., Beijing, China) was used in this study to measure the K value through the variable-head method. The test setup is shown in Figure 4. The calculation formula is as follows:

(10) $$K=2.3\frac{aL}{At}\mathrm{lg}\frac{h_{1}}{h_{2}},$$

where

a is the cross-sectional area of the variable-head pipe (in this test $a=1.075\;{\mathrm{cm}}^{2}$);

L is the length of the permeable path (in this test, L=4.0 m);

$h_{1}$ is the initial water head height;

$h_{2}$ is the final water head height;

A is the cross-sectional area of the specimen;

t is the time interval.

Permeability tests were conducted on the fault similar materials, and the permeability coefficients of specimens with typical mix ratios are shown in Table 4.

## 3. Analysis of Factors Affecting the Properties of Similar Materials

Through numerous mix ratio experiments, the influence of various components on the uniaxial compressive strength, cohesion, elastic modulus, and permeability coefficient, among other characteristic parameters of similar materials, has been investigated.

### 3.1. Analysis of Factors Affecting Uniaxial Compressive Strength

Among the components of similar materials, the binding capacity of the binder for coarse and fine aggregates directly reflects the strength of the material. The uniaxial compressive strength, as a key indicator of material strength, is influenced by the content of various components in the binder. Adjusting the gypsum and Vaseline content in fault similar materials significantly affects the uniaxial compressive strength of the material.

**Figure 5:**
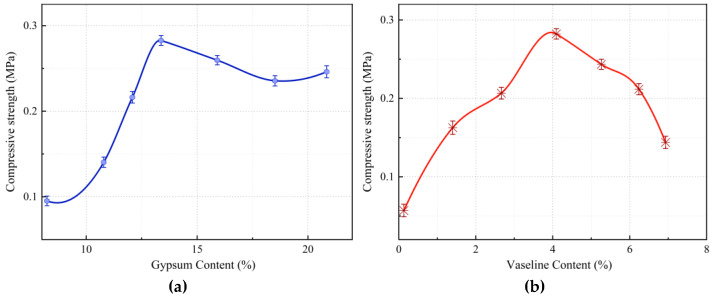
(**a**) The variation curve of compressive strength with gypsum content. (**b**) The variation curve of compressive strength with Vaseline content. Influence curves of gypsum and Vaseline contents on compressive strength of fault similar material.

As shown in Figure 5a, when the content of other components in the fault similar material remains constant, increasing the gypsum content leads to a corresponding increase in material strength. However, when the gypsum content exceeds 13%, strength begins to slightly decrease. Figure 5b illustrates that when the Vaseline content is less than 4%, the material strength increases with an increase in Vaseline content. However, when the Vaseline content exceeds 4%, the material strength decreases significantly. This is because at lower Vaseline contents, it acts as a partial binder, but as the content increases, Vaseline disrupts the bonding force between aggregates, leading to a reduction in material strength.

### 3.2. Analysis of Factors Affecting Permeability Coefficient

Permeability, as a key indicator of the hydro-physical properties of similar materials, is influenced by the material’s porosity and binder content. Varying the gypsum and Vaseline content in the material can significantly alter the permeability coefficient of the similar material.

**Figure 6:**
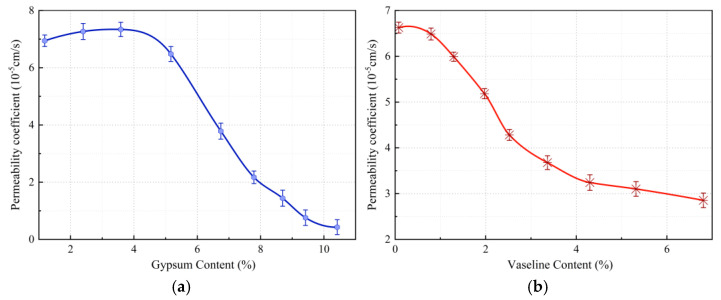
(**a**) The variation curve of the permeability coefficient with respect to gypsum content. (**b**) The variation curve of the permeability coefficient with respect to Vaseline content. Influence curves of gypsum and Vaseline contents on the permeability coefficient of fault similar materials.

Both gypsum and Vaseline content have a substantial impact on the permeability coefficient of fault similar materials. As shown in Figure 6a, when the gypsum content is below 4%, the permeability coefficient remains relatively stable. Although gypsum, as a binder, has some bonding effect on fine aggregates, its weak water-disintegration properties prevent a significant reduction in the permeability coefficient. When the gypsum content exceeds 4%, its cementing effect becomes more pronounced, resulting in a sharp decrease in the permeability coefficient. Figure 6b shows that the permeability coefficient decreases continuously with increasing Vaseline content and stabilizes at a value of 3.1 × 10^−5^ cm/s. This stabilization occurs because Vaseline, as a hydrophobic oil-based modifier, effectively fills the voids between coarse aggregates and blocks the percolation pathways when it reaches a certain content. Additionally, it can withstand some water pressure, leading to a final stable permeability coefficient.

### 3.3. Analysis of Factors Affecting Elastic Modulus

**Figure 7:**
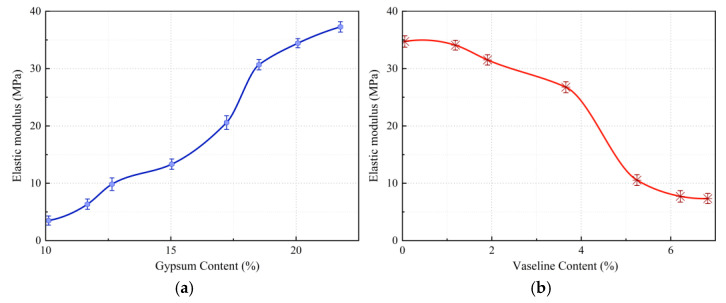
(**a**) The variation curve of elastic modulus with an increase in gypsum content. (**b**) The variation curve of elastic modulus with an increase in Vaseline content. Influence curves of gypsum and Vaseline contents on the elastic modulus of fault similar material.

The elastic modulus of fault similar materials can be adjusted by varying the gypsum and Vaseline content, as shown in Figure 7.

The elastic modulus of fault similar materials can be regulated by adjusting the gypsum and Vaseline content. As shown in Figure 7, the elastic modulus of fault similar materials increases approximately linearly with gypsum content and exhibits a negative correlation with Vaseline content. As depicted in Figure 7a, when the content of other components remains constant, increasing the gypsum content significantly improves the elastic modulus of the material. In contrast, Figure 7b shows that as the Vaseline content increases, the elastic modulus of the material continuously decreases.

### 3.4. Analysis of Influencing Factors of Cohesion

**Figure 8:**
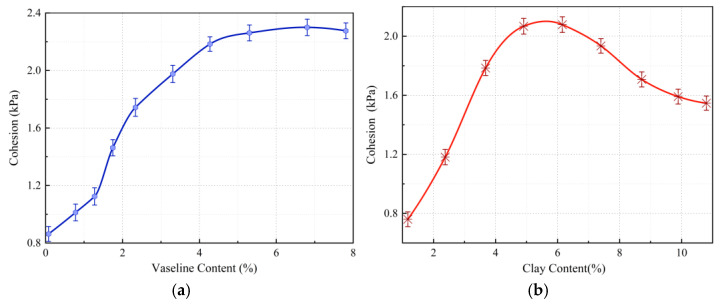
(**a**) The variation curve of cohesion with an increase in Vaseline content. (**b**) The variation curve of cohesion with an increase in clay content. Influence curves of Vaseline and clay contents on the cohesion of fault similar material.

The grading of aggregates and the binder content significantly affect the cohesion of similar materials. By varying Vaseline and clay contents, the cohesion of the material can be adjusted within a certain range, as shown in Figure 8.

Figure 8a illustrates the effect of Vaseline content on the cohesion of fault similar materials. Vaseline acts as an adjusting agent in fault materials, and when its content is below 4%, cohesion increases with increasing Vaseline content. However, when the Vaseline content exceeds 4%, the effect on cohesion becomes minimal, and it remains almost unchanged. Figure 8b shows the effect of clay content on the cohesion of fault materials. When the clay content is below 6%, the cohesion of the material increases as the clay content increases. However, when the clay content exceeds 6%, the cohesion begins to decrease, eventually stabilizing around 1.6 kPa.

Based on the above experimental analysis of the influence of similar material components and characteristic parameters, the regulatory mechanism of each component on the mechanical and permeability properties of the material can be clarified. For this reason, this study is based on the response relationships between the obtained components and parameters, which were obtained through the systematic proportioning test, and finally, the similar materials’ ratios were optimized in line with the target requirements shown in Table 5 below.

## 4. Application of the Tunnel Water Inrush Model Test

### 4.1. Test Overview

The total length of the Zhongjiashan Tunnel of Quannan Expressway in Jiangxi Province is about 5000 m, which is a deeply buried tunnel with a maximum burial depth of 450 m. There are several secondary faults, such as the major fault F2 and F3~F5 in the tunnel site area, among which F2 is the main fault, with a strike of N30° E, inclination of SE, a dip of 65°, and a width of 15–20 m. F3 and F5 are located at the southeast side of F2 at a distance of 50 m and 120 m, respectively.

**Figure 9:**
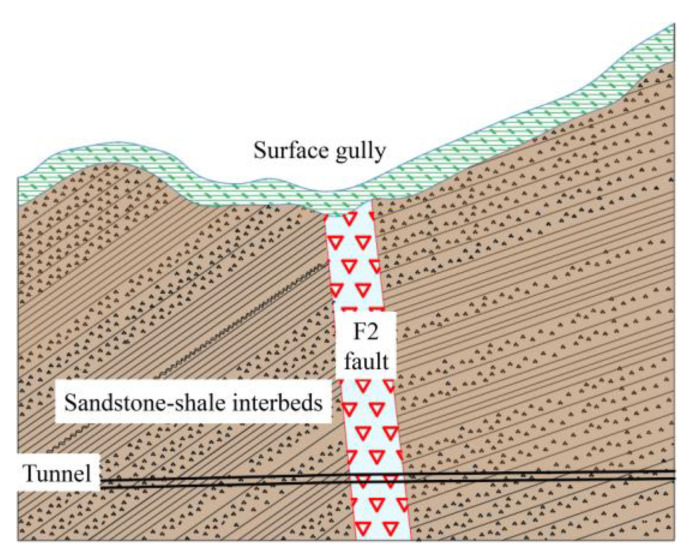
F2 fault profile.

The fault zone is rich in recharge water sources. Complex construction conditions are present, and water inrush disasters occur frequently. Construction at the F2 fault resulted in a number of large-scale water inrush disasters. In the F2 fault zone, the accumulation of broken gray-black carbonaceous sand shale is observed as a result of tectonic activities and the impact of the water inrush effect. The sediments show loose, broken characteristics and are accompanied by mud migration. The F2 fault section is shown in Figure 9.

The model test is based on the physical and mechanical parameters of the real fault rock samples of the project. The similarity ratio of the model test is selected as 1:60, and the target parameters of the fault similar material are calculated using the similarity criterion to determine the target parameters of the fault similar material. The evolution mechanism of the tunnel fault surge flooding is investigated. In order to verify the reasonableness of the developed material. The physical and mechanical parameters of the similar materials under the optimized ratios after the test are shown in Table 6, and by comparing the physical and mechanical parameters and hydrological parameters to those of the similar materials of the target expected faults, the results show that the materials can better simulate the key mechanical behaviors of the faulted rock body.

**Figure 10:**
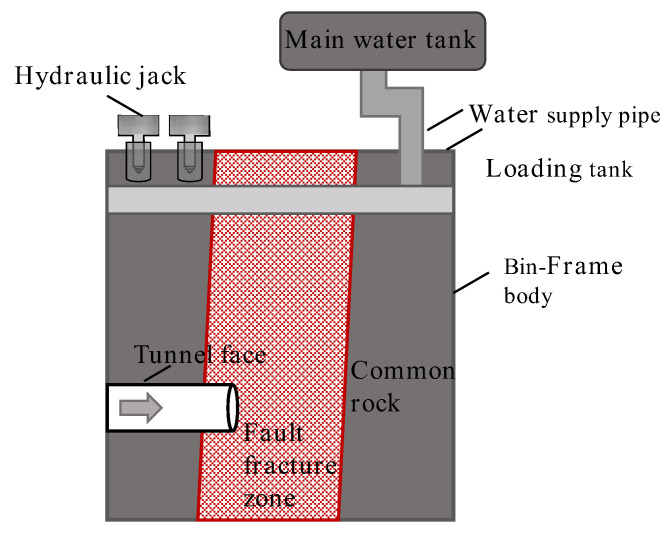
Model test system.

The prototype dimensions of the test are length × width × height = 96 m × 60 m × 180 m. Based on the scale ratio, the model dimensions are length × width × height = 1.6 m × 1.0 m × 3 m. The test system consists of four parts: the model frame, the geostress loading system, the water pressure loading system, and the information-monitoring system. The experimental model system is shown in Figure 10.

**Figure 11:**
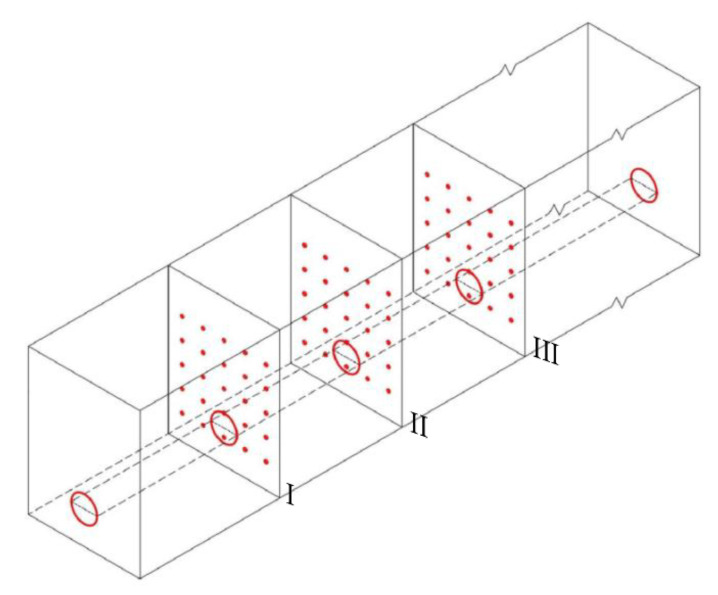
Layout of monitoring points.

The materials were prepared according to the specific mix ratios for fault similar materials, with the installation of components and material filling carried out simultaneously. Three monitoring sections were set within the fault: Section I, Section II, and Section III. Sections I and II were located within the fault zone, while Section III was positioned at the junction of the fault and the surrounding rock. Key areas, such as the tunnel crown, arch shoulders, waist, and invert, were equipped with sensors at each monitoring section. The specific layout of the monitoring sections and key sensor positions is shown in Figure 11.

### 4.2. Test Process and Results

Based on the determined material mix ratios, the individual raw material components were precisely weighed and mixed until uniform. The materials were then placed in layers using a partitioning method and compacted layer by layer, with compaction density being controlled to ensure uniformity. After completing the model’s filling, water was injected for 7 days to achieve saturation, and then, the tunnel simulation excavation was conducted until a water inrush disaster occurred. The excavation was performed using a stepping method, with a 2 cm advance per cycle and an 8 cm step length. After each excavation cycle, real-time monitoring of various information, including surrounding rock stress, displacement, and seepage pressure, was conducted. Once the data stabilized, the next excavation cycle could begin.

#### 4.2.1. Variation Law of Gushed Materials

**Figure 12:**
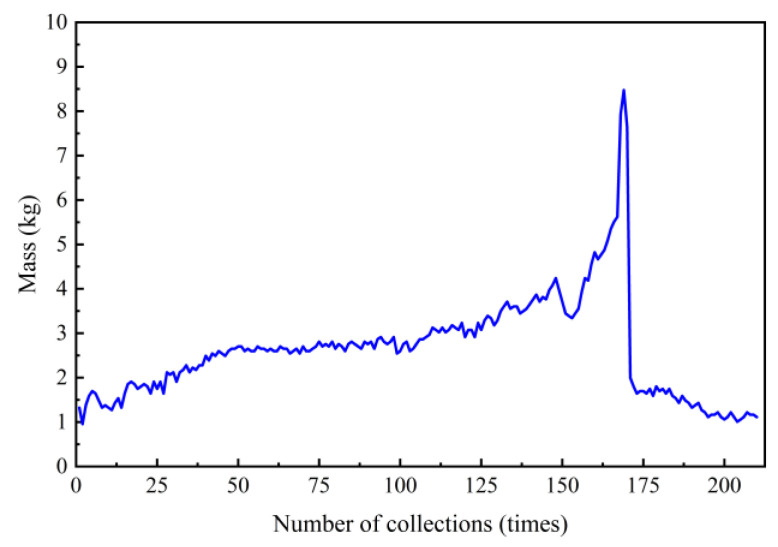
Outflow variation.

After exposing the fault, outflow data were collected approximately every 15 s. From Figure 12, the following observations can be made:

(1) Early Monitoring Stage: As seepage pressure increases, the weakened geotechnical body is carried out by groundwater, causing the surrounding rock porosity to gradually increase. The outflow mass shows an upward trend. Approximately 10 min after the start of monitoring, the outflow stabilizes. This is believed to be related to the stabilization of seepage pressure. When water inrush occurs, the outflow reaches its peak, indicating that a higher hydraulic gradient results in a more significant water inrush effect.
(2) Pre-Water Inrush Stage: Before the water inrush disaster occurs, starting from the 145th data collection point in Figure 12, the outflow mass decreases significantly. At this point, the structural integrity of the surrounding rock is damaged, and the geotechnical body is compacted. As a result, the pore volume of the material decreases, and the migration of soil particles is obstructed, leading to a reduction in outflow.
(3) Water Inrush Stage: As groundwater continues to penetrate and soften the surrounding rock, accompanied by a decrease in seepage pressure, the tunnel face begins to fail. A large volume of groundwater and solid matter flows into the tunnel, causing the outflow to suddenly increase, resulting in a water inrush disaster. After the disaster occurs, the outflow rate rapidly decreases and stabilizes. At this point, the changes in outflow are closely related to the water-disintegration characteristics of the fractured fault-zone rock and the magnitude of the seepage pressure.

#### 4.2.2. Displacement–Time Variation

To observe the impact of excavation disturbance on tunnel water inrush, a displacement meter was placed at Section III, where the fault intersects the normal surrounding rock. The position of this meter corresponds to monitoring point 5# in Sections I and II.

**Figure 13:**
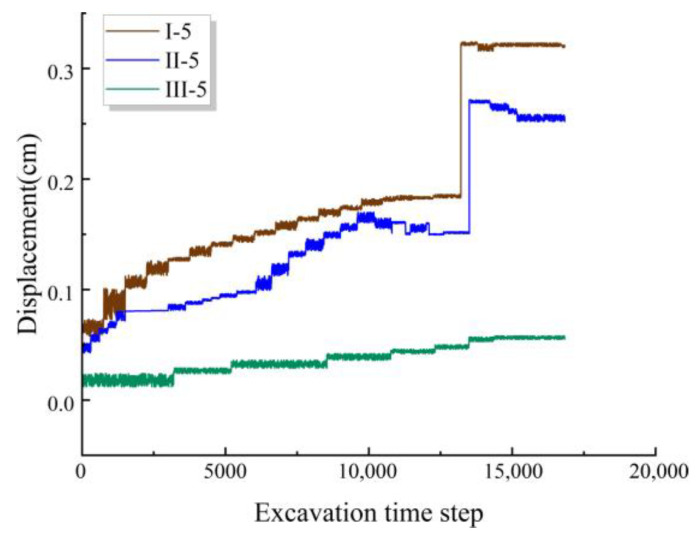
Horizontal displacement–time variation curve.

From Figure 13, it can be seen that prior to the occurrence of the disaster, all monitoring points show a slow increase in displacement. When the water inrush disaster occurs, there is a sudden surge in displacement at Sections I and II, while the displacement at the corresponding monitoring point in Section III remains relatively stable. Figure 13 indicates that, whether during normal surrounding rock excavation or after exposing the fault, the displacement amplitude of the fault-zone material is always greater than that of the normal surrounding rock. Moreover, the closer the monitoring point is to the fault-zone and tunnel excavation profile, the greater the displacement and settlement values. After the water inrush event concludes, the surrounding rock reaches a relatively stable state again.

### 4.3. Comparative Analysis of the Engineering Data

The following section presents a comparative analysis of the water inrush process, displacement data, and other relevant results from the Zhongjiashan Tunnel with respect to those from the model experiment.

#### 4.3.1. Water Inrush Process

**Figure 14:**
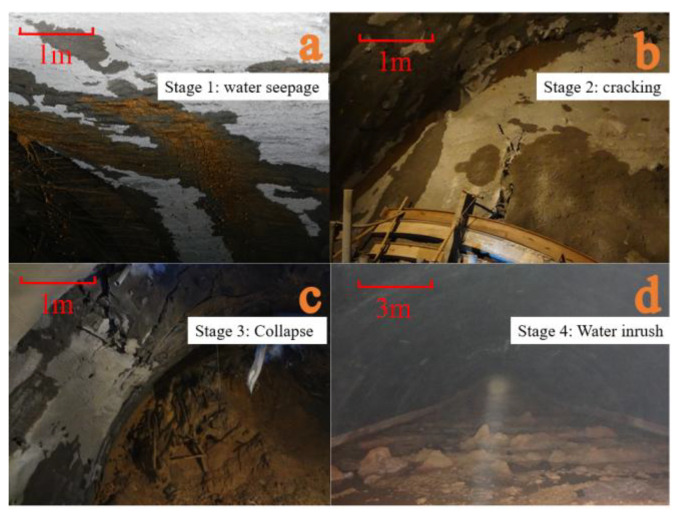
Water inrush process.

When the tunnel excavation reached the F2 fault zone, the excavation disturbance altered the groundwater seepage field. The process of tunnel water inrush is shown in Figure 14: In the left hole, K91 + 338, water seepage occurred on the right side of the tunnel vault (Figure 14a), and loose collapse occurred with a small gush of water (Figure 14b). This water discharge point became the breakthrough, with fault soil particles continuously carried out by the water’s flow (Figure 14c). Eventually, an irregular, circular water inrush hole with a diameter of approximately 1 m formed. Under high hydraulic head, a large volume of muddy water was ejected (Figure 14d), triggering a water inrush disaster. As the mud-filled rock mass and groundwater from the fault zone continued to flow out, the amount of muddy water gradually decreased to a natural outflow state. This process aligns well with the results from the model experiment.

#### 4.3.2. Displacement Change

**Figure 15:**
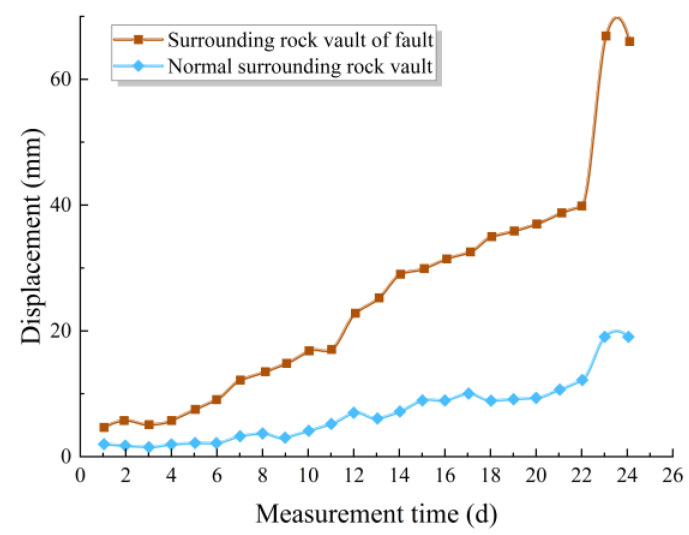
Actual displacement change curve of surrounding rock.

Figure 15 shows the displacement variation near the fault zone at the tunnel crown, both for the normal surrounding rock segment and the fault-zone rock after excavation through the F2 fault zone. It is evident that, at the same time, the displacement growth rate of the fault zone’s surrounding rock is faster than that of the normal surrounding rock. During the water inrush disaster, the displacement in the fault zone experienced a sharp increase, whereas the displacement at the crown of the normal surrounding rock was relatively unaffected by the disaster, only showing a slight increase during the water inrush event.

By comparing the model’s experimental results with the actual engineering water inrush process and related data, it is clear that the fault similar materials developed in this study can effectively simulate the fault water inrush process. The trends observed in the monitoring data are consistent with the physical laws governing multi-physical field changes. The experimental phenomena align closely with the actual engineering disaster process, confirming that the material meets the requirements of our experiment.

## 5. Conclusions

(1) A suitable similar material for fault water inrush model experiments was developed by optimizing the mix ratios. The material was composed of sand as the coarse aggregate; talc powder as the fine aggregate; gypsum and clay as binders; and Vaseline as an adjusting agent. This material effectively satisfies the simulation requirements for both the physical–mechanical properties and hydro-physical characteristics of faults.
(2) The uniaxial compressive strength and permeability coefficient of the similar material are primarily controlled by the content of gypsum and Vaseline. Increasing gypsum contents enhances strength, but excessive amounts reduce permeability. When Vaseline contents are less than 4%, strength is increased, but an excess of Vaseline disrupts the bonding structure. Cohesion is mainly influenced by the content of clay and Vaseline. When clay contents are below 6%, cohesion increases with the amount of clay, but it decreases when the content exceeds 6%. Vaseline significantly enhances cohesion when present in low concentrations.
(3) The similar material was applied in a three-dimensional model experiment simulating tunnel fault water inrush disasters. The experiment successfully reproduced the disaster evolution process of fault water inrush, and the monitoring data from the experiment showed good consistency with actual engineering disaster phenomena. This demonstrates that the similar material’s physical–mechanical properties and hydro-physical characteristics are well-suited for simulating fault water inrush processes and provides a reliable foundation for studying the response mechanisms of various physical fields during the fault water inrush event.
(4) There are still limitations in the development of similar materials and the simulation of tunnel fault surge flooding. The optimization of the ratio of similar materials is based on a specific fault prototype, and its adaptability to different surrounding rock conditions or complex hydrogeological environments still needs to be further verified. In the future, we can combine multidisciplinary methods to improve the material’s constitutive model and expand the conditions of multi-field coupling tests to reveal the disaster mechanism of fault water inrush in a more comprehensive way.

## Figures and Tables

**Table 1:** Physical–mechanical parameters of faulted protoliths and targeted similar materials.

Material Type	Density ρ/(g/cm^3^)	Compressive Strength $\sigma_{c}$/MPa	Elastic Modulus E/GPa	Permeability Coefficient K/(cm/s)	Cohesion c/kPa	Internal Friction Angle φ/(°)
Fault undisturbed rock mass	1.94~2.05	8.0~12.0	1.0~1.2	2.46 × 10^−4^~5.76 × 10^−4^	118.53~159.78	33~41
Targeted Similar Materials	1.94~2.05	0.13~0.2	0.017~0.02	3.16 × 10^−5^~7.44 × 10^−5^	1.98~2.66	33~41

**Table 2:** Values of c and φ of fault similar materials’ representative specimens.

Test Number	Material Ratio (S:T:C:G:W:V)	Cohesion c (kPa)	Angle of Internal Friction φ (°)
1	1:0.05:0.04:0.16:0.12:0.09	2.208	32
2	1:0.05:0.04:0.30:0.12:0.09	2.672	37
3	1:0.05:0.04:0.25:0.12:0.09	2.262	35
4	1:0.05:0.01:0.25:0.12:0.09	2.036	40
5	1:0.05:0.05:0.25:0.12:0.09	1.943	36

Note: S:T:C:G:W:V represents sand, talcum powder, clay, gypsum, water, and Vaseline, respectively.

**Table 3:** Disintegration time of fault similar material specimens with typical mix ratios.

Test Number	Material Ratio (S:T:C:G:W:V)	Disintegration Time (s)
1	1:0.24:0.05:0.25:0.15:0.12	185
2	1:0.24:0.05:0.32:0.15:0.12	215
3	1:0.24:0.02:0.25:0.15:0.12	330

**Table 4:** Permeability coefficients of fault similar material specimens with typical mix ratios.

Test Number	Material Ratio (S:T:G:C:V:W)	Permeability Coefficient K (cm/s)
1	1:0.24:0.05:0.25:0.15:0.12	8.4 × 10^−5^
2	1:0.24:0.05:0.32:0.15:0.12	5.2 × 10^−5^
3	1:0.24:0.05:0.25:0.15:0.12	4.5 × 10^−5^

**Table 5:** Fault similar materials’ mix ratios.

A:B	S:G	W:G	T:C	W:V
3.51:1	6.22:1	0.82:1	2.3:1	3.52:1

**Table 6:** Comparison of physical and mechanical parameters of materials.

Medium	Density ρ/(g/cm^3^)	Compressive Strength $\sigma_{c}$/MPa	Elastic Modulus E/GPa	Permeability Coefficient K/(cm/s)	Cohesion c/kPa	Internal Friction Angle φ/(°)
Test proportioning materials	1.98	0.18	0.02	3.87 × 10^−5^	2	38

## Data Availability

The original contributions presented in this study are included in the article. Further inquiries can be directed to the corresponding author.

## References

[1] Zhang Q., Jiang Q., Zhang X., Wang D. (2019). Model test on development characteristics and displacement variation of water and mud inrush on tunnel in fault fracture zone. Nat. Hazards.

[2] Shi S., Xie X., Bu L., Li L., Zhou Z. (2018). Hazard-based evaluation model of water inrush disaster sources in karst tunnels and its engineering application. Environ. Earth Sci..

[3] Dai C., Long Y., Lv Y., Hou W., Sui H. (2019). Water inrush mechanism and safety control in drilling and blasting construction of subsea tunnel. J. Coast. Res..

[4] Song T., Zeng J., Ma J., Ma C., Li T., Xia T., Aloisio A. (2021). Water inrush risk assessment based on AHP and advance forecast approach, A case study in the Micangshan tunnel. Adv. Civ. Eng..

[5] Wang M., Yang W., Zhou Z., Yang J., Yang F., Sheng S. (2022). Experimental study on fractal characteristics of fault filling medium in the tunnel and relationship between fractal dimension and permeability coefficient. Geomech. Geophys. Geo-Energy Geo-Resour..

[6] Wang X., Zhang L., Zhang Q., Liu R., Huang C. (2025). A stepwise calculation method for grouting penetration in rough rock fracture based on fracture segment division. Tunn. Undergr. Space Technol..

[7] Xin G., Wang B., Zheng H., Zeng L., Yang X. (2024). Study on Water Inrush Characteristics of Hard Rock Tunnel Crossing Heterogeneous Faults. Appl. Sci..

[8] Li X., Xue Y., Zhang Z. (2023). Progressive evolution model of fault water inrush caused by underground excavation based on multiphysical fields. Geofluids.

[9] Zhou Z., Li L., Shi S., Liu C., Gao C., Tu W., Wang M. (2020). Study on tunnel water inrush mechanism and simulation of seepage failure process. Rock Soil Mech..

[10] Zhong Z.-L., Shen Z., Qiao H.-Y., Li Y.-P., Zhu K.-X. (2025). Study on mechanism of water and mud inrush in deep-buried large-section tunnel crossing water-rich fault fracture zone. Rock Mech. Rock Eng..

[11] Li Y., Weng X., Wang R., Zhang L., Zhang L. (2020). Experimental study on mechanism of water and mud inrush in tunnel crossing fault fracture zone. J. Highw. Transp. Res. Dev..

[12] Zhang Q., Chen W., Yuan J., Liu Q., Rong C. (2020). Experimental study on evolution characteristics of water and mud inrush in fault fractured zones. Rock Soil Mech..

[13] Wang Y., Chen F., Sui W., Meng F., Geng F. (2022). Large-scale model test for studying the water inrush during tunnel excavation in fault. Bull. Eng. Geol. Environ..

[14] Dai S.H., Wang H.R., Han R.J., Wang Z.W. (2020). Properties of similar materials used in fluid-solid coupling model test. Rock Soil Mech..

[15] Ko T.Y., Lee S.S. (2020). Characteristics of crack growth in rock-like materials under monotonic and cyclic loading conditions. Appl. Sci..

[16] Zan W., Lai J., Zhang W., Yang Q., Qin Y., Su X. (2024). Experimental and applied research on similar materials to granular mixtures for solid-liquid coupling model test of an underwater tunnel. Constr. Build. Mater..

[17] Shemenda A.I. (1992). Horizontal lithosphere compression and subduction: Constraints provided by physical modeling. J. Geophys. Res. Solid Earth.

[18] Zhang Z., Zhang Q., Xiang W., Yin X., Xue T., Lin H., Lei C., Xin G. (2021). Development and application of new-style hydro-mechanical coupling similar materials in complex environment. J. Cent. South Univ. (Sci. Technol.).

[19] Xu Z., Luo Y., Chen J., Su Z., Zhu T., Yuan J. (2021). Mechanical properties and reasonable proportioning of similar materials in physical model test of tunnel lining cracking. Constr. Build. Mater..

[20] Liu S., Liu W. (2018). Experimental development process of a new fluid–solid coupling similar-material based on the orthogonal test. Processes.

[21] Guo Y., Yang Y., Kong Z., He J., Wu H. (2022). Development of Similar Materials for Liquid-Solid Coupling and Its Application in Water Outburst and Mud Outburst Model Test of Deep Tunnel. Geofluids.

[22] Wu W., Guo J., Liu X., Zhu Z., Wang E. (2023). Experimental study on similar materials for fluid-solid coupling for model test of water inrush in karst tunnel. Geotech. Geol. Eng..

[23] Ning Z., Li J., Wang H., Li Z., Zhuang D., Xu W., Zambrano-Narvaez G., Zhan L., Chen Y. (2025). Application of physical model test in underground engineering: A review of methods and technologies. Transp. Geotech..

[24] Gibson R.E. (1974). The analytical method in soil mechanics. Geotechnique.

[25] Ren X., Xu G., Chen Z., Ran S., Zhang J. (2024). Development of similar materials for fluid-solid coupling model testing and application in damage constitutive models. Sci. Rep..

